# Avaliação dos efeitos do subgalato de bismuto na proliferação de miofibroblastos

**DOI:** 10.1590/1677-5449.009515

**Published:** 2016

**Authors:** Rubianne Ligório de Lima, Cláudia Paraguaçu Sampaio, Karin Caroline Seidel, Melina Branco, Rafaela Mabile Sobreiro

**Affiliations:** 1 Pontifícia Universidade Católica do Paraná – PUC-PR, Faculdade de Medicina, Curitiba, PR, Brasil.

**Keywords:** cicatrização, miofibroblastos, otolaringologia

## Abstract

**Contexto:**

O subgalato de bismuto é um metal pesado e insolúvel, utilizado por suas propriedades adstringentes e hemostáticas.

**Objetivo:**

Avaliar os efeitos do subgalato de bismuto na cicatrização mediante observação de miofibroblastos em pele de ratos.

**Métodos:**

Foram utilizados 60 ratos da linhagem Wistar, que receberam uma ferida no dorso da pele. Os animais foram divididos em dois grupos: controle (aplicação diária de cloreto de sódio a 0,9%) e experimental (aplicação diária de 0,5 mg de subgalato de bismuto). Cada grupo foi subdividido em três subgrupos, que foram reoperados para retirada da ferida em 3, 7 e 14 dias. Foi realizada coloração de hematoxilina eosina, picrosirius e imuno-histoquímica para avaliar contagem de miofibroblastos, resposta inflamatória e síntese de colágeno.

**Resultados:**

Não foi encontrada diferença entre os grupos controle e experimento com relação ao processo inflamatório – subgrupos 3 dias (p = 1), 7 dias (p = 0,474) e 14 dias (p = 303). A avaliação dos colágenos tipo I e III no grupo-controle não demonstrou benefícios de cicatrização – 3 dias (p = 0,436), 7 dias (p = 0,853) e 14 dias (p = 0,436); já no grupo experimental, houve aumento dos colágenos tipos I e III nos subgrupos 3 e 14 dias (p = 0,005). A imuno-histoquímica confirmou os resultados encontrados na coloração hematoxilina eosina, na qual a área de miofibroblastos entre os subgrupos, nos grupos experimental (p = 0,4) e controle (p = 0,336), foi indiferente.

**Conclusão:**

A utilização do subgalato de bismuto em ferida de pele de ratos não evidenciou benefícios na cicatrização, ou seja, não houve diferença na fibroplasia quando comparados os grupos experimental e controle.

## INTRODUÇÃO

O subgalato de bismuto é uma substância de coloração amarelada que se apresenta na forma de pó inodoro e sofre descoloração na presença de luz solar^1^. Vem sendo cada vez mais utilizado pelos profissionais relacionados à otorrinolaringologia e odontologia devido às suas propriedades adstringentes e hemostáticas. Entre as formas utilizadas estão o tratamento tópico de feridas abertas, o tratamento de úlceras gastroduodenais, como antidiarreico, para controle do odor em colostomias, em cirurgias odontológicas, manejo da epistaxe e, empiricamente, em adenotonsilectomias^1^^-^ 3.

As maiores preocupações dos otorrinolaringologistas quanto a uma amidalectomia é diminuir o sangramento do transoperatório, reduzir o tempo cirúrgico e evitar as complicações pós-operatórias; ou seja, busca-se um procedimento seguro^1^^-^ 3.

O subgalato de bismuto, metal pesado e relativamente insolúvel em água, apresenta qualidades adstringentes (que ativam o fator XII da cascata de coagulação), acelera a formação do coágulo sanguíneo e melhora a hemostasia^4^. Frente a isso, há uma necessidade de estudos controlados e randomizados, metodologicamente aceitos, para dar melhor sustento à sua utilização.

Alguns serviços de otorrinolaringologia não utilizam o subgalato de bismuto, alegando que o resultado do seu uso pós-operatório imediato não revela benefício, enquanto outros referem grande facilidade no controle de sangramento. Os trabalhos, de uma forma geral, enfocam seu uso referindo controle de sangramento pós-operatório imediato.

A literatura não é objetiva quanto ao benefício do subgalato na hemostasia ou no efeito cicatricial, havendo discordâncias. Além disso, não trabalha os possíveis efeitos pró-cicatriciais, deixando a desejar em se tratando de grupos comparativos ou controle.

Como a fibroplasia é uma das importantes fases cicatriciais, se o subgalato de bismuto induzir uma maior atividade fibroplásica, espera-se ter uma maior quantidade de miofibroblastos, os quais levariam a um processo de contração precoce, otimizando a cicatrização.

Os fibroblastos são os responsáveis pela síntese, remodelação e deposição da matriz, além de interagirem com a mesma. As moléculas estruturais que formam a nova matriz extracelular contribuem para a formação do tecido de granulação, o qual servirá de base para a migração celular. A formação do tecido de granulação começa próximo ao terceiro dia^5^.

O fibroblasto excreta um monômero denominado pró-colágeno. As fibras reticulares do colágeno tipo III são mais delgadas que as do colágeno tipo I e possuem maior quantidade de carboidratos. A cicatrização das feridas é realizada inicialmente com o colágeno tipo III, que mais tarde será substituído pelo colágeno tipo I, mais resistente^5^.

O objetivo geral deste artigo foi avaliar se o subgalato de bismuto promove interferência em alguma das fases da cicatrização por meio de análise histológica e imuno-histoquímica e pela observação do desenvolvimento de miofibroblastos nas feridas em pele do dorso. Sendo um material de fácil manejo (pó) e custo reduzido, seu uso poderia beneficiar a população do ponto de vista cicatricial, caso os estudos indiquem essa correlação.

## MÉTODOS

Após aprovação do Comitê de Ética no Uso de Animais (CEUA) (protocolo 780) e de acordo com as recomendações do Colégio Brasileiro em Experimentação Animal (COBEA), os experimentos foram realizados no Laboratório de Técnica Operatória e Cirurgia Experimental da Pontifícia Universidade Católica do Paraná (PUC-PR) no mês de julho de 2013.

Foram utilizados 60 ratos machos (*Rattus norvegicus albinus, Rodentia mammalia*) da linhagem Wistar, adultos jovens com idade de 110 dias e com peso médio entre 250 g e 300 g, provenientes do Biotério Central. Os animais foram mantidos com alimentação *ad libitum* e água. Os mesmos ratos deste estudo foram utilizados em conjunto com o projeto “Avaliação dos efeitos do subgalato de bismuto na angiogênese: estudo experimental em ratos”.

No Laboratório de Técnica Operatória e Cirurgia Experimental, os animais foram anestesiados com 0,1 ml/100 g de peso do animal com uma mistura de 1 ml de ketamina (50 mg) com 1 ml de xilazina 2% (20 mg) por via intramuscular na porção posterior da coxa. Em seguida, foram posicionados em decúbito ventral, em um suporte de madeira, sendo fixados os membros anteriores e os posteriores. Foi realizada tricotomia na região dorsal de cada animal, em área de 24 cm^2^ (seis centímetros de comprimento por quatro centímetros de largura), localizada a partir de uma linha imaginária traçada entre os membros anteriores, estendendo-se por seis centímetros em direção caudal. Logo depois, foi realizada antissepsia com polivinilpirrolidona-iodo e delimitação da área operatória com campo esterilizado fenestrado.

No centro da área tricotomizada, foi feita uma demarcação na pele de cada rato por rotação da borda cortante de um *punch* metálico, com dois centímetros de diâmetro. Foi ressecado o segmento de pele circular, de acordo com a demarcação, sendo aprofundada a incisão até expor a fáscia muscular dorsal.

Após o término do ato operatório, os animais receberam diclofenaco de potássio na dose de 10 mg/kg, por via intramuscular, com finalidade analgésica e anti-inflamatória. Os animais foram encaminhados de volta ao Biotério Central.

Os animais foram aleatoriamente marcados e divididos. Foram três grupos experimentais e três grupos-controle. Cada grupo era composto por 10 animais. Os animais dos grupos experimentais foram submetidos a aplicação diária de 0,5 mg de subgalato de bismuto em suas feridas. Os grupos-controle tiveram suas feridas tratadas diariamente com solução de cloreto de sódio a 0,9%, conforme preconiza a literatura. A eutanásia ocorreu de acordo com os grupos 3, 7 e 14 dias de tratamento.

Os animais foram reoperados para a retirada da ferida de acordo com o seu grupo. Cada ato operatório foi realizado após a devida anestesia do animal (como descrito anteriormente). O fragmento foi retirado de cada ferida cirúrgica, com margem de um centímetro de pele íntegra em torno da lesão, com profundidade até a musculatura dorsal do rato.

Imediatamente após a reoperação de cada animal, foi realizada eutanásia por dose letal de tiopental sódico intraperitonial (120 mg/kg), sendo este o método de eutanásia recomendado para roedores e outros pequenos mamíferos, contido na Resolução 714 do Conselho Federal de Medicina Veterinária de 20 de junho de 2002.

Não houve nenhuma perda de animal e material durante a realização da pesquisa.

Os segmentos destinados à histologia, após retirados do dorso do rato, foram estendidos sobre papel-cartão identificado. Na sequência, foram mergulhados em recipiente com formol a 10% por 24 horas, sendo cortados e colocados em cassetes para a montagem dos blocos de parafina. As lâminas foram coradas com hematoxilina eosina (HE) e picrosirius, e analisadas em microscópio óptico.

Na HE, foi realizada a contagem de células monomorfonucleares e polimorfonucleares e de vasos para a caracterização da fase do processo inflamatório de acordo com a seguinte classificação: Tabelas 1 e 2 6.

**Tabela 1:** Contagem celular do processo inflamatório.

**Número de células**	**Polimorfonucleares**	**Monomorfonucleares**
Até 50	–1	1
50-100	–2	2
> 100	–3	3

**Tabela 2:** Caracterização da fase do processo inflamatório de acordo com o escore final de cada grupo.

**Fase do processo inflamatório**	**Escore final de classificação**
Agudo	–9 a –3
Subagudo	–2,9 a 3
Crônico	3 a 9

Inicialmente, para cada momento de avaliação (3 dias, 7 dias e 14 dias), foi comparada a fase do processo inflamatório nos dois grupos, controle e experimental. Em seguida, considerando-se cada grupo, os momentos de comparação foram feitos dois a dois. Testou-se a hipótese nula de que a distribuição sobre as classificações da fase do processo inflamatório é igual nos dois momentos de avaliação comparados *versus* a hipótese alternativa de distribuições diferentes.

A coloração picrosirius avaliou o colágeno através da birrefringência específica de cada tipo de colágeno, fazendo uso de um microscópio de luz polarizada. Com relação ao percentual de área de colágeno, a comparação dos grupos em cada momento de avaliação (3 dias, 7 dias e 14 dias) foi feita através do teste não paramétrico de Mann-Whitney. A comparação dos momentos de avaliação considerando-se cada grupo foi feita através do teste não paramétrico de Kruskal-Wallis. Para a análise comparativa dos grupos e dos momentos de avaliação em relação à fase do processo inflamatório, foi usado o teste exato de Fisher. Valores de p < 0,05 indicaram significância estatística. Os dados foram analisados com o programa computacional IBM SPSS Statistics v.20.

O material também foi encaminhado para processamento imuno-histoquímico, visando pesquisa com alfa-SMA, Fator 8 e CD34. Porém, apenas no alfa-SMA foi realizada a leitura, pois o Fator 8 e o CD34 apresentaram pouca especificidade com relação à coloração de musculatura lisa analisada.

Após a análise histológica e imuno-histoquímica, foi realizada análise estatística com métodos paramétricos e não paramétricos, sendo então elaborada a documentação final e o artigo para posterior publicação.

A análise dos resultados relativos à imuno-histoquímica foi feita em cima da área corada em castanho, que evidencia a área expressa pelos miofibroblastos, e da contagem de vasos para avaliação da angiogênese.

## RESULTADOS

### Avaliação do processo inflamatório com contagem de células monomorfonucleares e polimorfonucleares

**Figura 1:**
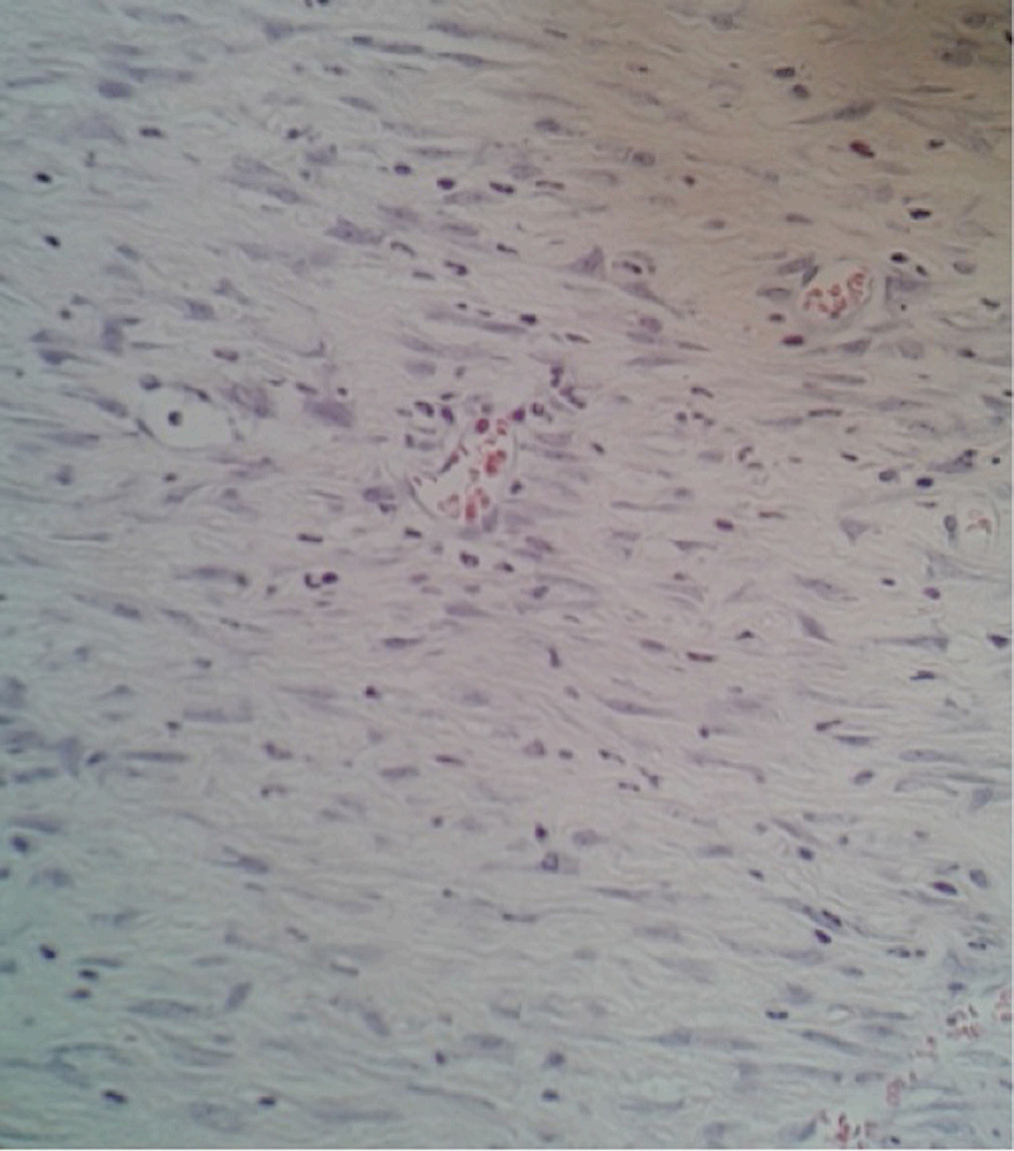
Coloração hematoxilina eosina no grupo-controle/subgrupo 14 dias.

Nas duas avaliações iniciais, com relação ao processo inflamatório, não houve diferença de cicatrização nos respectivos grupos – subgrupos 3 dias (p = 1), 7 dias (p = 0,474) e 14 dias (p = 0,303) (Figuras 1 e 2).

**Figura 2:**
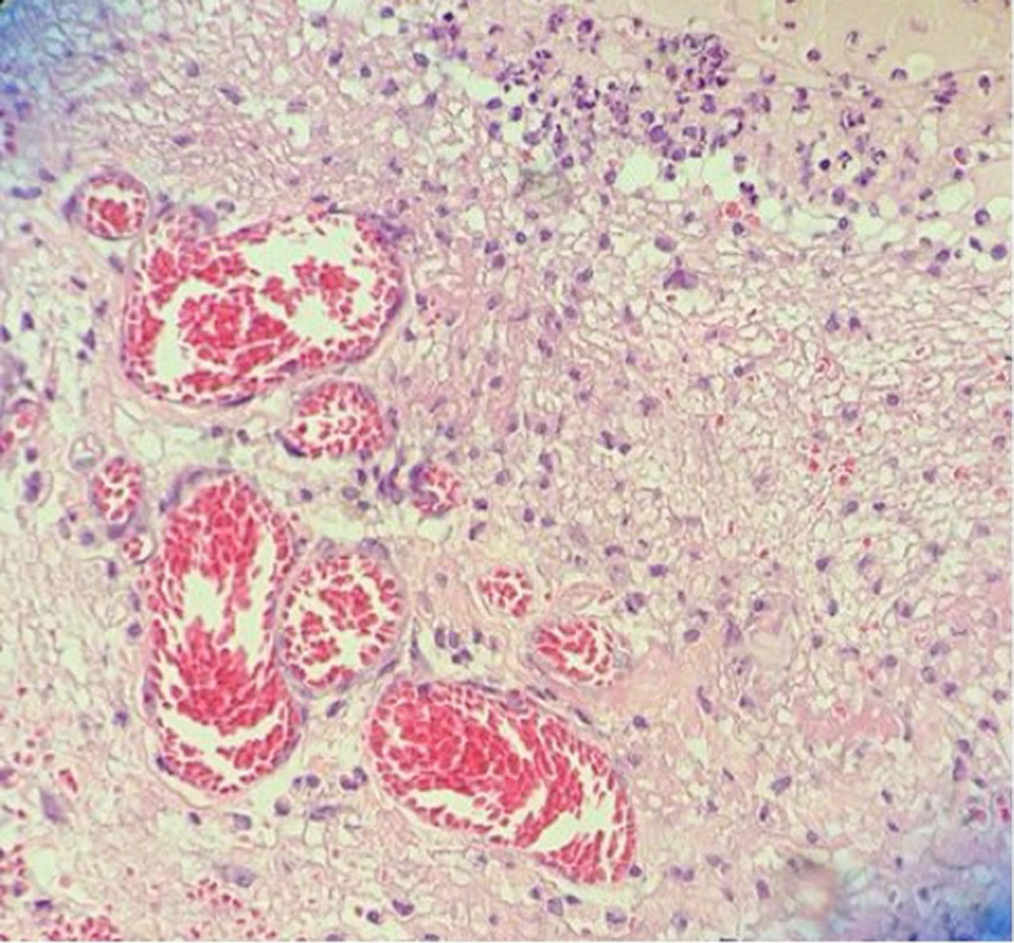
Coloração hematoxilina eosina no grupo experimental/subgrupo 14 dias.

### Avaliação do colágeno através da coloração picrosirius

**Figura 3:**
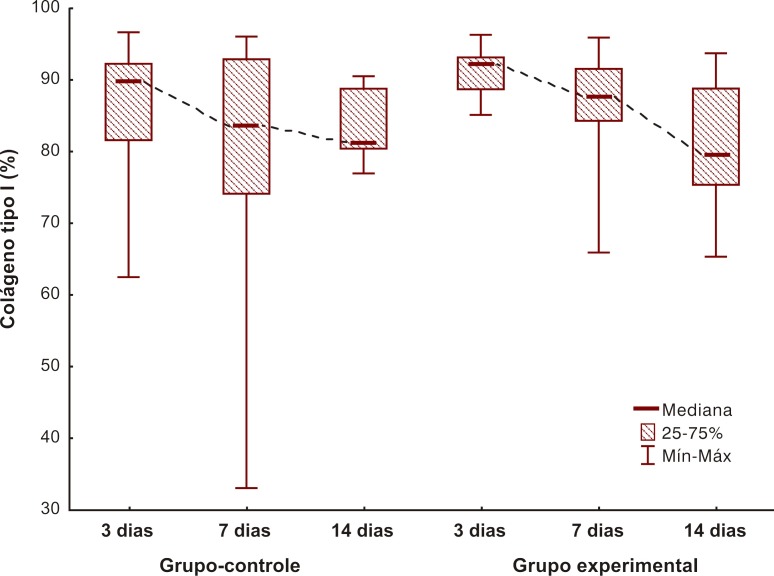
Comparação dos grupos com relação ao colágeno maduro.

A avaliação do colágeno tipo I foi feito entre os grupos experimental e controle, bem como intragrupos, comparando os dias entre si. A produção de colágeno tipo I foi semelhante entre os grupos experimental e controle, ou seja, não houve diferença quantitativa de colágeno tipo I entre os grupos (p = 0,330). Já no grupo experimental, houve aumento importante da produção de colágeno tipo I entre 3 e 14 dias, fato não observado no controle (p = 0,024) (Figura 3).

**Figura 4:**
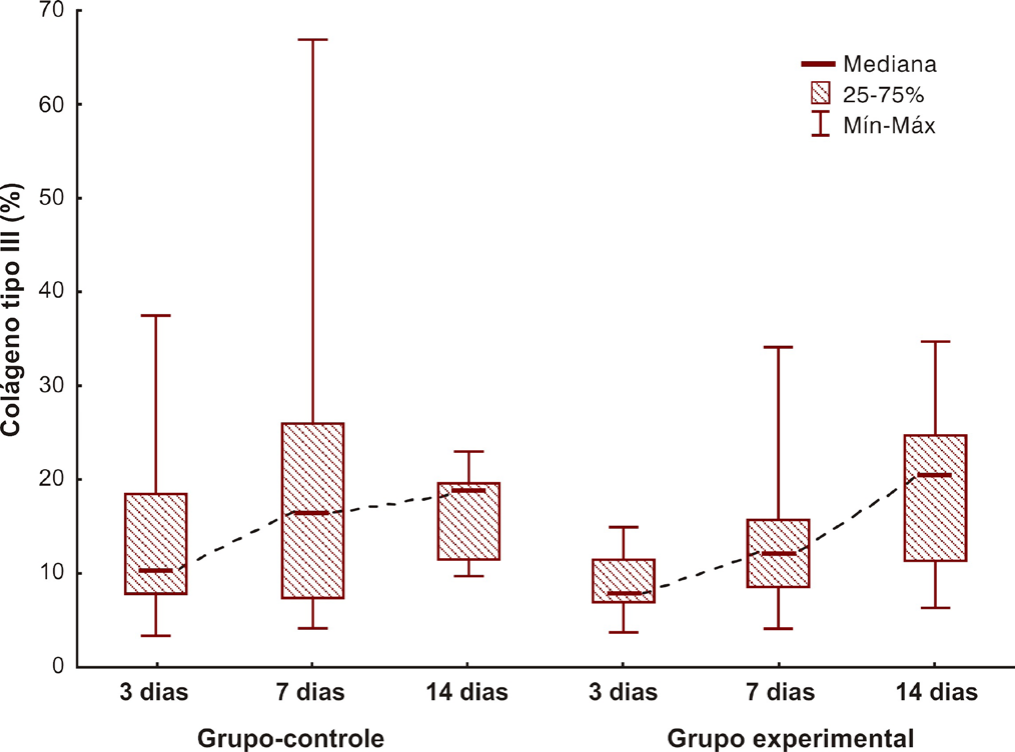
Comparação dos grupos com relação ao colágeno imaturo.

**Figura 5:**
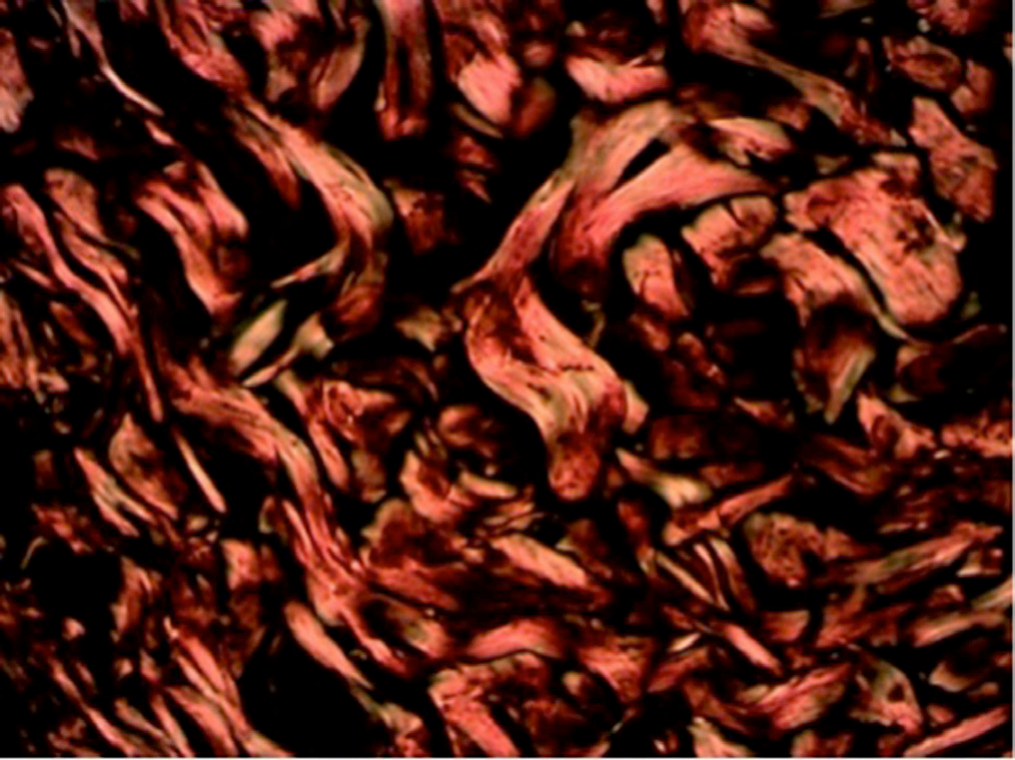
Coloração picrosirius no grupo-controle/subgrupo 14 dias.

Com relação aos resultados apresentados no colágeno tipo III, cuja análise foi realizada da mesma maneira que a análise do colágeno tipo I, os resultados foram também os mesmos (grupo-controle: p = 0,330; e grupo experimental: p = 0,024) (Figuras 4-5 6).

**Figura 6:**
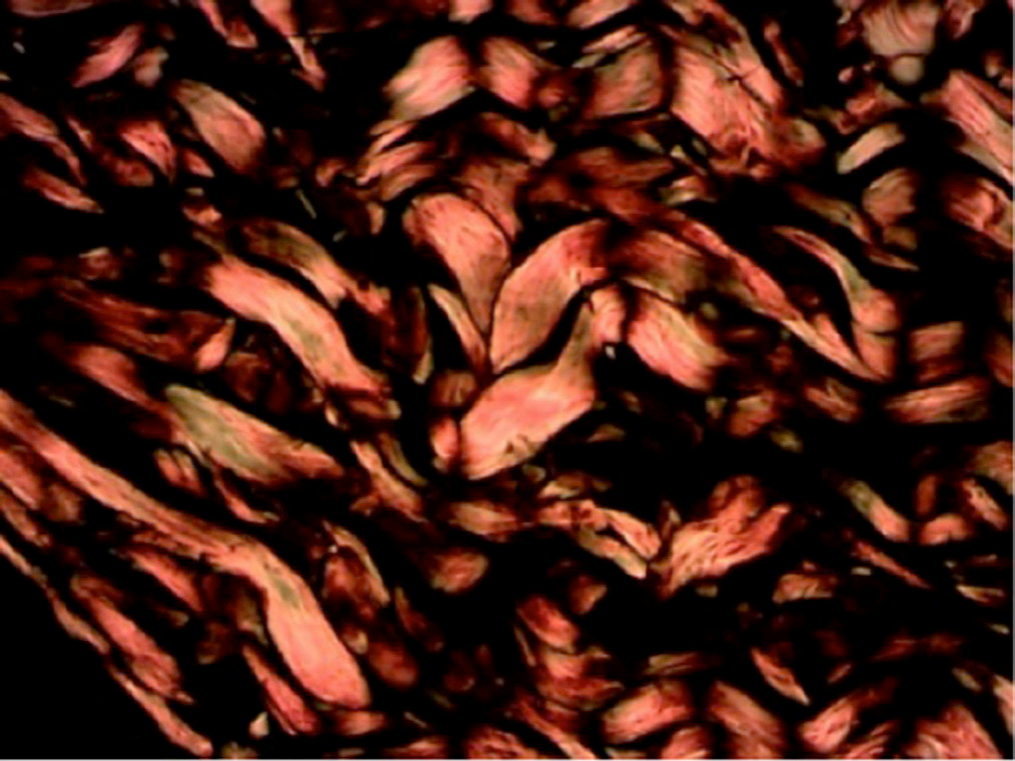
Coloração picrosirius no grupo experimental/subgrupo 14 dias.

### Processamento imuno-histoquímico com pesquisa de alfa-SMA

**Figura 7:**
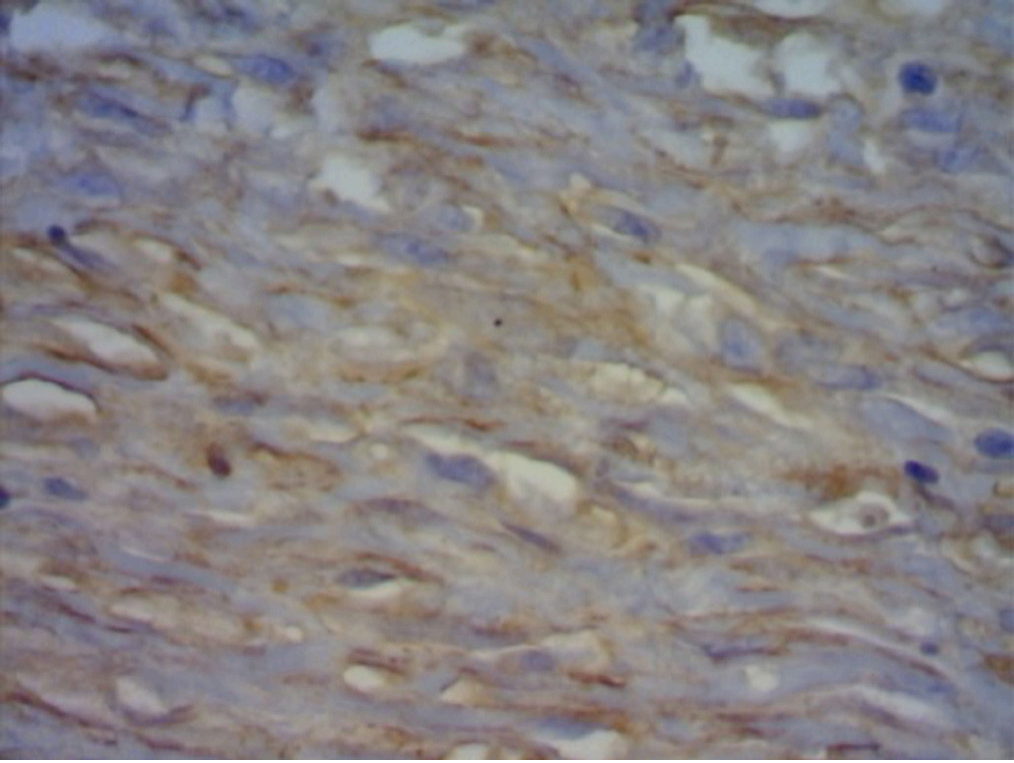
Imuno-histoquímica no grupo-controle/subgrupo 14 dias.

Na análise dos miofibroblastos, não houve diferença na fase cicatricial, bem como na análise dos vasos para a angiogênese (grupo controle: p = 0,336; e grupo experimental: p = 0,400) (Figuras 7 e 8).

**Figura 8:**
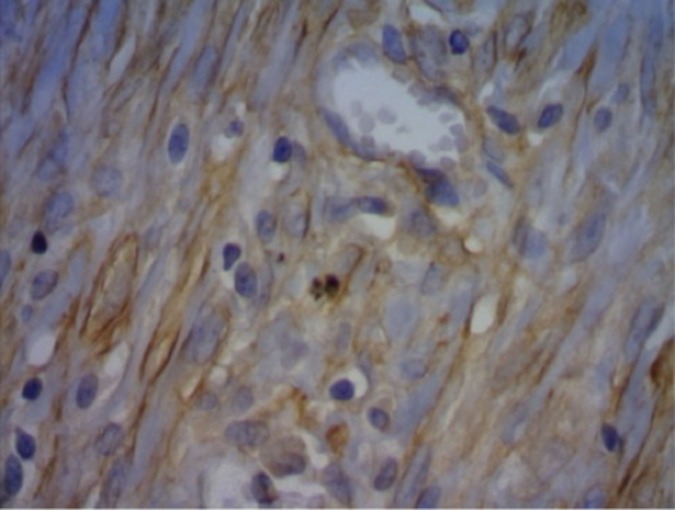
Imuno-histoquímica no grupo experimental/subgrupo 14 dias.

## DISCUSSÃO

Atualmente, estão sendo investigados métodos com o objetivo de diminuir e evitar a hemorragia trans e pós-operatória em adenoamigdalectomias. Por esse motivo, vários meios têm sido comparados. O método por criocirurgia é considerado inviável pelo seu alto custo e pela dificuldade de manipulação e armazenamento do nitrogênio líquido. Já o método a *laser* necessita de uma correta potência do raio, além de tempo de exposição e ângulo de enfoque precisos, para que a técnica seja promissora, com possibilidade de danos às estruturas adjacentes devido à alta temperatura do raio (750 a 900 °C)^7^.

Devido a essas dificuldades apresentadas pelos outros métodos, mesmo sem estudos que nos indiquem a real eficácia do subgalato de bismuto, este tem sido um método aceitável e muito difundido em todo o mundo como agente hemostático em operações de amígdalas.

A literatura não trabalha possíveis efeitos pró-cicatriciais do uso do subgalato de bismuto. Para obter melhores opções hemostáticas, buscamos compreender melhor as qualidades cicatricionais dessa substância.

Outros autores obtiveram excelentes resultados hemostáticos na área cruenta deixada após tonsilectomia palatina com uso de tampão de gaze embebido em solução de subgalato de bismuto a 100%. Além disso, foi considerado um método de baixo custo e fácil realização, tendo sido rara a necessidade de sutura ou ligadura dos vasos da loja^8^.

Quando comparamos o grau de inflamação dos grupos experimental e controle, notamos que não há diferença significativa. O mesmo foi observado em um estudo que avaliou o processo inflamatório do uso de subgalato de bismuto em hepatectomias em ratos^9^.

Outro estudo que avaliou a cicatrização também em dorso de ratos Wistar, porém utilizando períodos diferentes para os subgrupos (1, 4, 7, 11 e 18 dias), também concluiu que o subgalato de bismuto não interferiu na qualidade do processo de reparação, sendo biocompatível com os tecidos. Ele apresentou uma área maior de tecido de granulação pela presença física do material, podendo ser indicado como hemostático, sem efeito significativo no processo de reparação, o que demonstra que o efeito independe do tempo de aplicação da substância^10^.

No presente estudo, não foi visto benefício do uso da solução de subgalato de bismuto na cicatrização da pele, bem como na fibroplasia. Um estudo mostrou retardo na cicatrização e na fibroplasia em mucosas, mesmo com a diluição e a quantidade aplicada da substância em estudo sendo as mesmas^11^.

Uma possível razão para a diferença de comportamento do subgalato de bismuto em mucosas e na pele são as propriedades individuais de cada um desses tecidos. Em comparação com a pele, a mucosa oral apresenta menor quantidade de tecido cicatricial, tanto sob aspecto clínico como histológico, por apresentar menor quantidade de fibras. Em termos de formação de tecido cicatricial, as diferenças entre a mucosa e a pele são vantajosas, tanto sob aspecto estético como funcional. Pesquisadores acreditam que essas diferenças devam estar relacionadas às origens embrionárias dos fibroblastos dos dois tecidos. Os fibroblastos da pele derivam do mesoderma, e os da mucosa oral, de células da crista neural^12^.

A menor quantidade de fibras provenientes da crista neural pode ter permitido maior grau de absorção do subgalato, o qual, em função dessa maior quantidade absorvida, pode ter apresentado efeito tóxico, alterando a fibroplasia e, consequentemente, a cicatrização de forma geral.

Na pele, a maior quantidade de fibras provenientes do mesoderma pode ter servido de barreira, impedindo maior absorção e resultando em menor efeito tóxico. Não houve interferência na fibroplasia, e a cicatrização foi mantida sem alterações.

Com a realização da coloração picrosirius, foi evidenciado, no grupo experimental, um aumento importante da produção de colágeno tipo I entre 3 e 14 dias, fato não observado no controle (p = 0,024). Esse resultado não foi encontrado na imuno-histoquímica. Uma possível razão para tal fato coloca em questionamento a efetividade do marcador, uma vez que ele não é específico para ratos. Nesse caso, seria válido realizar novamente a pesquisa utilizando outro marcador para confirmar a imuno-histoquímica, mas, em função de custo e dificuldade, optou-se por não realizar outro marcador neste momento, ficando o bloco de parafina reservado para outras pesquisas.

## CONCLUSÃO

A utilização do subgalato de bismuto em ferida de pele de ratos não evidenciou benefícios relativos à proliferação de miofibroblastos quando comparados os grupos experimental e controle.
