# Supplementary material for: Avaliação dos efeitos do subgalato de bismuto na proliferação de miofibroblastos
Source: J Vasc Bras. 2016 Jul-Sep;15(3):210–6. [Article in Portuguese] doi: 10.1590/1677-5449.009515 (PMC5829758; doi:10.1590/1677-5449.009515)
Supplement: Translation

# Translation: Assessment of the effects of bismuth subgallate on proliferation of myofibroblasts: an experimental study in rats

**Authors:** Rubianne Ligório de Lima, Cláudia Paraguaçu Sampaio, Karin Caroline Seidel, Melina Branco, Rafaela Mabile Sobreiro

**Affiliations:** 1 Pontifícia Universidade Católica do Paraná – PUC-PR, Faculdade de Medicina, Curitiba, PR, Brazil.

**Keywords:** wound healing, myofibroblasts, otolaryngology

## Abstract

**Background:**

Bismuth subgallate is an insoluble heavy metal that is used for its astringent and hemostatic properties.

**Objective:**

To evaluate the effects of bismuth subgallate on the healing process by observation of myofibroblasts in the skin of rats.

**Methods:**

A sample of 60 Wistar rats was used. Each rat was subjected to a dorsal skin wound and allocated to one of two groups: a control group, in which 0.9% sodium chloride was administered daily, or an experimental group, in which 0.5 mg of bismuth subgallate was administered daily. Each of these groups was further subdivided into three subsets, which were reoperated after 3, 7 and 14 days respectively for excision and collection of the skin wound specimens. Samples were treated with hematoxylin eosin, picrosirius, and immunohistochemical staining to enable assessment of myofibroblast counts, inflammatory response phase, and collagen synthesis.

**Results:**

No inflammatory process differences were detected between the control and experimental groups at 3 days (p = 1), 7 days (p = 0.474), or 14 days (p = 303). Evaluation of types I and III collagen in the control group did not demonstrate healing benefits at 3 days (p = 0.436), 7 days (p = 0.853), or 14 days (p = 0.436); whereas in the experimental group there were increases in types I and III collagen at 3 and 14 days (p = 0.005). Immunohistochemical analysis confirmed the results of hematoxylin eosin staining, since there were no differences between subsets in terms of area of myofibroblasts, in the experimental (p = 0.4) or the control (p = 0.336) groups.

**Conclusions:**

Administration of bismuth subgallate to skin wounds in rats did not result in any evidence of benefits to healing, i.e., no difference in fibroplasia was detected when experimental and control groups were compared.

## INTRODUCTION

Bismuth subgallate is a yellowish substance that is presented in the form of an odorless powder and which undergoes discoloration in the presence of sunlight.^1^ It is increasingly being used by professionals working in otorhinolaryngology and dentistry because of its astringent and hemostatic properties. Applications include topical treatment of open wounds, treatment of gastroduodenal ulcers, as an antidiarrheal agent, to control colostomy odor, during dental surgery, for management of epistaxis and, empirically, in adenotonsillectomies.^1^^-^ 3

When performing an amygdalectomy, the otorhinolaryngologist’s primary concerns are to reduce transoperative bleeding, reduce duration of surgery, and avoid postoperative complications; in other words, to achieve a safe procedure.^1^^-^ 3

Bismuth subgallate is a heavy metal that is relatively insoluble in water and has astringent properties (via activation of factor XII of the coagulation cascade), accelerates formation of blood clots, and improves hemostasis.^4^ In view of its increasing use, there is a need for controlled and randomized studies with acceptable methodology to provide better support for its use.

Some otorhinolaryngology services do not employ bismuth subgallate, claiming that its administration during the immediate postoperative period is not beneficial, whereas others describe it as very useful for controlling bleeding. In general, studies have focused on its use to control bleeding during the immediate postoperative period.

There is no objective answer in the literature with relation to the benefits of subgallate for hemostasis or for healing and studies are contradictory. Additionally, its possible beneficial effects on healing have not been investigated and there is room for improvement in terms of use of comparison or control groups.

Fibroplasia is an important phase in healing and if bismuth subgallate does induce increased fibroplastic activity, it is to be expected that the number of myofibroblasts will be increases, leading to an early contraction process, optimizing healing.

Fibroblasts are responsible for synthesis, remodeling, and deposition of the matrix and also interact with the matrix. The structural molecules that make up the new extracellular matrix contribute to formation of granulation tissue, which in turn provides a foundation for cell migration. Formation of granulation tissue begins around the third day.^5^

Fibroblasts secrete a monomer called procollagen. The reticular fibers of type III collagen are narrower than those of type I collagen and have larger quantities of carbohydrates. Wound healing initiates with type III collagen, which will later be substituted by the more resistant type I collagen.^5^

The overall objective of this article was to evaluate whether bismuth subgallate has effects on some of the phases of healing by histological and immunohistochemical analysis and by observation of myofibroblast development in dorsal skin wounds. Since it is a material that is easy to handle (in powder form) and is inexpensive, its use in healing could benefit the population, if studies indicate that such a correlation does exist.

## METHODS

After approval was granted by the local Animal Research Ethics Committee (protocol 780), and in accordance with Brazilian College of Animal Experimentation (COBEA) recommendations, the experiments were conducted at the Experimental Surgery and Operating Techniques Laboratory at the Pontifícia Universidade Católica do Paraná (PUC-PR) during July of 2013.

A sample of 60 male Wistar rats (*Rattus norvegicus albinus, Rodentia mammalia*), all young adults aged 110 days and with mean weight from 250 g to 300 g, were obtained from the University’s Central Animal House. These animals were provided with food and water ad libitum. The same sample of rats was also used in a project entitled “Evaluation of the effects of bismuth subgallate on angiogenesis: experimental study with rats”.

At the Experimental Surgery and Operating Techniques Laboratory, the animals were anesthetized with 0.1 ml/100 g of body weight with a mixture of 1 ml of ketamine (50 mg) with 1 ml of 2% xylazine (20 mg) administered intramuscularly to the posterior thigh. They were then positioned in decubitus ventral on a wooden support to which front and hind legs were fixed. An area of 24 cm^2^ (6 centimeters long by 4 centimeters wide) was shaved on the dorsal region of each animal, located with relation to an imaginary line between the front limbs and extending 6 centimeters in the caudal direction. Shortly afterwards, antisepsis was performed with povidone iodine and the operating are was delimited with a fenestrated sterile field.

In the center of the shaved area, a demarcation with a diameter of 2 centimeters was performed on the skin of each rat by rotating the cutting edge of a metal punch. This circular section of skin was resected following the demarcation, and the incision was taken to a depth sufficient to expose the dorsal muscle fascia.

After the operation, each animal was given intramuscular potassium diclofenac at a dosage of 10 mg/kg with the objective of achieving analgesia and preventing inflammation. The animals were transported back to the Central Animal House.

The animals were marked and separated at random to form three experimental groups and three control groups of 10 animals each. Animals in experimental groups were administered 0.5 mg of bismuth subgallate to their wounds daily. The wounds of control animals were treated daily with 0.9% sodium chloride solution, as recommended in the literature. One control group and one experimental group each underwent euthanasia on day 3, day 7, and day 14.

The animals were reoperated to remove the wound specimens on the days scheduled for each group. Each operation was performed after anesthesia of the animal (as described above). The specimen removed included a margin of 1 cm of intact skin around each surgical wound and was to the depth of the dorsal musculature.

Immediately after reoperation, each animal was euthanized with a lethal dose of intraperitonial sodium thiopental (120 mg/kg), which is the euthanasia method for rodents and other small mammals recommended in Federal Veterinary Medical Council Resolution 714 of June 20, 2002.

None of the animals or specimens were lost during the study.

After removal from the rats, the histology specimens were laid out on labeled cards. They were then immersed in a receptacle containing 10% formol for 24 hours and were then cut and placed in cassettes for mounting of the paraffin blocks. Slides were stained with hematoxylin eosin (HE) and picrosirius and analyzed under an optical microscope.

Monomorphonuclear and polymorphonuclear cells and vessels were counted on the HE slides to characterize the inflammatory process, which was scored as shown in Table 1 and then classified as shown in Table 2.^6^

**Table 1:** Scores for inflammatory process cell counts.

**Number of cells**	**Polymorphonuclear**	**Monomorphonuclear**
Up to 50	–1	1
50-100	–2	2
> 100	–3	3

**Table 2:** Classification of inflammatory process phases by final score for each group.

**Inflammatory process score**	**Final classification score**
Acute	–9 to –3
Subacute	–2.9 to 3
Chronic	3 to 9

Initially, inflammatory process phases were compared between the two groups, control and experimental, for each of the three assessment points (3 days, 7 days, and 14 days). Next, within each group, the assessment points were compared with each other, two by two. The null hypothesis that the distribution of inflammatory process phases was equal at the two different assessment times was tested against the alternative hypothesis that the distributions were different.

Picrosirius staining was used to assess collagen by means of the birefringence specific to each type of collagen, using a polarized light microscope. The Mann-Whitney nonparametric test was used to compare the percentage collagen area in each group at each of the assessment times (3 days, 7 days, and 14 days). Intra-group comparisons of different assessment times against each other were performed using the Kruskal-Wallis nonparametric test. Fisher’s exact test was used for the comparative analyses of groups and assessment times in terms of inflammatory process phases. P values < 0.05 were considered indicative of statistical significance. Data were analyzed with IBM SPSS Statistics v.20.

Specimens were also sent for immunohistochemical processing, with the intention of testing for alpha-SMA, Factor 8 and CD34. However, readings were only conducted for alpha-SMA, because the smooth muscle stain analyzed provided very little specificity for Factor 8 and CD34.

After histological and immunohistochemical analysis, statistical analysis was conducted using parametric and nonparametric methods, before compilation of the final documentation and writing of the article for publication.

Analysis of immunohistochemical results was based on the area stained a chestnut color, which indicates the area of myofibroblast expression, and on counting vessels to evaluate angiogenesis.

## RESULTS

### Evaluation of the inflammatory process by monomorphonuclear and polymorphonuclear cell counts

The two initial analyses of the inflammatory process revealed no differences in healing between the respective groups at 3 days (p = 1), 7 days (p = 0.474), or 14 days (p = 0.303) (Figures 1 and 2).

**Figure 1:** Hematoxylin eosin staining for control group/14 day subset.

**Figure 2:** Hematoxylin eosin staining for experimental group/14 day subset.

### Evaluation of collagen by picrosirius staining

Type I collagen was compared between experimental and control groups and intra-group analyses were conducted comparing the three different days against each other. Production of type I collagen in the experimental and control groups was similar, i.e. there was no quantitative difference in type I collagen between the groups (p = 0.330). In the experimental group, there was a considerable increase in type I collagen from 3 to 14 days, which was not observed in the control group (p = 0.024) (Figure 3).

**Figure 3:** Comparison of groups for mature collagen.

Analysis of the results for type III collagen was performed in the same manner as analysis of type I collagen results and revealed the same results (control group: p = 0.330; and experimental group: p = 0.024) (Figures 4-5 6).

**Figure 4:** Comparison of groups for immature collagen.

**Figure 5:** Picrosirius staining for control group/14 day subset.

**Figure 6:** Picrosirius staining for experimental group/14 day subset.

### Immunohistochemical processing with alpha-SMA analysis

The analysis of myofibroblasts did not reveal any differences in terms of healing phase and analysis of vessels revealed no difference in angiogenesis (control group: p = 0.336; and experimental group: p = 0.400) (Figures 7 and 8).

**Figure 7:** Immunohistochemical staining for control group/14 day subset.

**Figure 8:** Immunohistochemical staining for experimental group/14 day subset.

## DISCUSSION

Methods are currently being investigated that are intended to reduce and avoid transoperative and postoperative hemorrhages in adenotonsillectomies and several different approaches have been compared. Methods based on cryosurgery are considered unfeasible because of the high cost and the difficulties with handling and storage of liquid nitrogen. In turn, methods employing lasers demand the correct power beam, in addition to precise exposure time and focus angles if a technique is to be considered promising, and there is the possibility of damage to adjacent structures because of the high temperatures of the beam (750 to 900 °C).^7^

These problems related to other methods have resulted in worldwide acceptance and adoption of bismuth subgallate as a hemostatic agent for tonsil surgery, even in the absence of studies demonstrating its true efficacy.

The literature does not cover the possible benefits for healing of bismuth subgallate. With the intention of contributing to better options for hemostasis, we attempted to increase understanding of this substance’s healing properties.

Other authors reported excellent hemostatic results in the area of bleeding left by tonsillectomy using a gauze dressing soaked in a 100% solution of bismuth subgallate. The method was also considered low cost and easily employed, and it is rare that suture or ligature of the vessels in the region is needed.^8^

When we compared the degree of inflammation between the experimental and control groups, we noted that there was no significant difference. The same was observed in a study that investigated the inflammatory process with use of bismuth subgallate in partial hepatectomies in rats.^9^

Another study assessed healing in the dorsal area of Wistar rats, but using different periods for the subsets (1, 4, 7, 11, and 18 days), and also concluded that bismuth subgallate did not impact on the quality of the healing process and was biocompatible with the tissues. That study observed a larger area of granulation tissue, due to the physical presence of the material, concluding that it can be prescribed as a hemostatic with no significant effect on the healing process, which demonstrates that this finding is independent of the duration of administration of the substance.^10^

In the present study, no beneficial effect on skin healing from use of the bismuth subgallate solution was observed and there was also no effect on fibroplasia. A different study observed delayed healing and fibroplasia in mucosas, even though the dilution and quantity of the substance administered were the same in both studies.^11^

One possible reason for the difference in the behavior of bismuth subgallate in mucosas and in skin is the individual properties of each of these tissue types. Compared with skin, oral mucosa exhibits smaller quantities of scar tissue, both clinically and histologically, because it has fewer fibers. In terms of formation of scar tissue, the differences between mucosa and skin are advantageous, both from esthetic and functional perspectives. Researchers believe that these differences are related to the embryonic origins of the fibroblasts in the two types of tissue. The fibroblasts in skin are derived from the mesoderm, and those in oral mucosa from cells of the neural crest.^12^

The smaller quantity of fibers originating from the neural crest may enable a higher degree of absorption of the subgallate, which may have had toxic effects because of the greater quantity absorbed, altering fibroplasia and, consequently, healing in general.

In the skin, the larger quantity of fibers originating from the mesoderm may have created a barrier, impeding greater absorption and resulting in reduced toxicity. There was no effect on fibroplasia, and healing was unaltered.

The picrosirius staining revealed a considerable increase in production of type I collagen from 3 to 14 days in the experimental group, which was not observed in the control group (p = 0.024). The immunohistochemical analysis did not detect this result. One possible explanation for this fact calls into question the effectiveness of the marker employed, since it is not specific for rats. In this case it would be interesting to conduct the test again with a different marker in order to confirm the immunohistochemical results, but, because of the costs and difficulties involved, the decision was taken not to use a different marker at this point and the paraffin blocks have been stored for future studies.

## CONCLUSIONS

In rats, treating skin wounds with bismuth subgallate did not exhibit benefits in terms of myofibroblast proliferation when experimental and control groups were compared.
